# Active motif finder - a bio-tool based on mutational structures in DNA sequences

**DOI:** 10.1016/S1674-8301(11)60059-6

**Published:** 2011-11

**Authors:** Mani Udayakumar, Palaniyandi Shanmuga-priya, Kamalakannan Hemavathi, Rengasamy Seenivasagam

**Affiliations:** a Department of Bioinformatics;; b Department of Bioinformatics;; c Department of Bioinformatics, School of Chemical and Biotechnology, Shanmugha Arts Science Technology & Research Academy (SASTRA University), Tanjore, Tamilnadu 613402, India;; d Division of Drug Discovery and Development, Centre of Molecular and Computational Biology, Department of Botany, St. Joseph College, Bangalore, Karnataka 560027, India.

**Keywords:** mutations, alignment score matrix, back track, indels, pattern occurrence, DNA sequences.

## Abstract

Active Motif Finder (AMF) is a novel algorithmic tool, designed based on mutations in DNA sequences. Tools available at present for finding motifs are based on matching a given motif in the query sequence. AMF describes a new algorithm that identifies the occurrences of patterns which possess all kinds of mutations like insertion, deletion and mismatch. The algorithm is mainly based on the Alignment Score Matrix (ASM) computation by comparing input motif with full length sequence. Much of the effort in bioinformatics is directed to identify these motifs in the sequences of newly discovered genes. The proposed bio-tool serves as an open resource for analysis and useful for studying polymorphisms in DNA sequences. AMF can be searched *via* a user-friendly interface. This tool is intended to serve the scientific community working in the areas of chemical and structural biology, and is freely available to all users, at http://www.sastra.edu/scbt/amf/.

## INTRODUCTION

Motif discovery from a set of sequences is a very important problem in biology. Although numerous studies have been done on computational techniques for sequence motif discovery, discovering motifs in a large number of sequences still remains challenging[1]. Motif is a particular amino-acid sequence that is characteristic of a specific biochemical function. Motifs can often be recognized by simple inspection of the amino-acid sequence of a protein, and, when detected, provide strong evidence for biochemical function. Sequence motifs always have functional implications, and are becoming increasingly important in the analysis of gene regulation. They are short, recurring patterns in DNA that are presumed to have a biological function. Often they indicate sequence-specific binding sites for proteins such as nucleases and transcription factors (TF). Others are involved in important processes at the RNA level, including ribosome binding, mRNA processing (splicing, editing, and polyadenylation) and transcription termination[2]. Frequent chemical changes in the residues of the DNA motifs result in mutation which affects the biological functions. Active Motif Finder(AMF) is basically designed with a new approach to identify motifs in a given sequence with any number of mutations (insertions, deletions or mismatches) in it. This would help to give a clear idea about the changes that evolution has brought about in them[1].

To this end, there are a limited range of web servers and tools available for researchers to analyze the information on polymorphisms and mutations in DNA structures; they are, W-ChIPMotifs[3], Motif Tool Manager[4], GIMSAN (GIbbsMarkov with Significance ANalysis)[5], HeliCis[6], fREDUCE[7], DRIM[8], SMOTIF[9], MOST[10] and MULTIPROFILER algorithm[11]. Although the above-mentioned resources have efficient algorithms in motif discovery, they are not based on polymorphisms and mutations and also very limited in motif searching patterns. The online resources, W-ChIPMotifs, Motif Tool Manager, GIMSAN and HeliCis web application tools are little complicated and time consuming based on ChIP-developed high-throughput data and incorporated various *ab initio* motif discovery tools such as MEME, MaMF, and Weeder for *de novo* motif discovery. The package fREDUCE is less sensitive to degenerate motifs. The recently released tool DRIM is highly complex for identifying regulatory sequence elements in a variety of applications ranging from expression and ChIP-chip to CpG methylation data. SMOTIF is faster, but only applicable for simple motifs. MOST web tool is based on a multi-step approach and is appropriate for seeded motifs only. The package MULTIPROFILER algorithm is assigned for subtle motifs only. Appreciably, here, we present a novel algorithmic tool which is AMF for significance in mutation based analysis of DNA sequences.

## MATERIALS AND METHODS

AMF predicts the motifs based on the number of mutations. The description of algorithm is composed of two main components: required input and output. The first option provides a simple yet powerful user query interface, which aids users in searching for mutation based motif with three inputs. The input options are explained in “AMF help” portion. They are partitioned as: 1) minimum of a protein/DNA sequence in which motifs are to be found out (a user must enter the sequences in fasta format one after another, and the sample file was also given); 2) motif pattern that is to be searched for; 3) the maximum number of mutations allowed. The mutation is entered as a numerical ranging from 0, 1, 2, 3……*n*. Based on the mutations entered, the server predicts the motif in the sequences. AMF also has the capabilities of introducing gaps between the sequences for finding the motifs. If no mutations are entered, then the exact pattern is searched in the sequences. The second component contains the following information: 1) motif starting and ending indicated by the symbol of “*”; 2) motifs are highlighted with colors as: (a) “Red” color indicates exact match, (b) “_” indicates deletion, (c) “Blue” color indicates mismatch, (d) “Green” color indicates insertion, (e) “Black” color indicates not in the motif; 3) motif positions (a) with 0 mutation, (b) with 1 mutation, (c) with 2 mutations, and (d) with 3 mutations. The “AMF Demonstration” option in the tool interface provides absolute information about tool's working procedure.

AMF works by using a different strategy than the usual string techniques used to identify motifs (pattern) in a query sequence (long string). This is because by using the string techniques, it is possible only to identify motifs that exactly match and those with some mismatches in them. But in order to find motifs with mutations like insertion and deletion, this process would become very complex and tedious to implement. To find motifs with insertions, the motif should be extended temporarily at that point and in case of deletion, the motif should be shortened. In order to avoid these complexities, AMF has been developed, which works based on the construction of alignment matrix between the motif and the query sequence, which is similar to those used in Smith-Waterman and Needleman-Wunsch algorithms. It uses the techniques of both local and global alignment. The alignment matrix is initialized using the concepts similar to local alignment where alignment starts at each position and is independent of the previous scores. Whereas the calculations that follow use the concepts of global alignment where the scores are retained as the actual ones unlike local alignment (where negative values are replaced by zeroes). The reward and penalty scores for match (2), mismatch (-1), deletion (-1) and insertion (-3) are awarded for the construction of the alignment matrix. After the construction of the alignment matrix, backtracking is used to get the alignments.

AMF takes three input boxes, one for the main sequence “seq”, next for the pattern “pat” and the last for an integer input “N” that specifies the maximum number of mutations that one wishes to have in a pattern. Thus, the program takes these inputs and gives the desired results i.e. all different occurrences of pattern starting from 0 mutation (exact matches) to the N mutation occurrences as specified in input. The approach to the problem is through the alignment matrix score of pattern with the main string. But the scoring methods and weightings are different. The output contains all the information about each occurrence and all the mutations are labelled with different colors. Finally, the positions of all occurrences are also given.

## RESULTS

### Study 1: Alignment matrix with different mutations

**Fig. 1:**
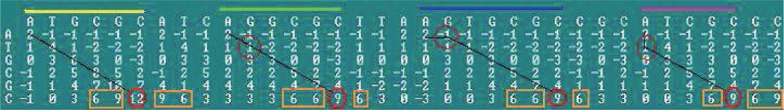
Alignment matrix with different mutations. Yellow-matching; Green-mismatch; Blue-insertion; Pinkdeletion.

Alignment matrix with different mutations is shown in ***Fig. 1***. Here yellow – matching; green – mismatch; blue – insertion; pink - deletion.

**Figure:**
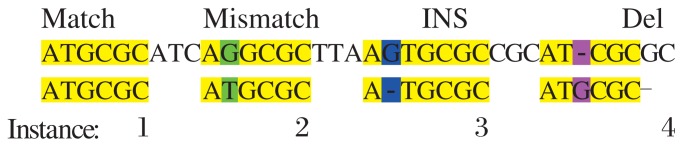


Calculation of scores for individual instances using normal values [match: 2 and for all others: -1] will be as follows.

**Figure:**
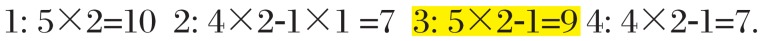


For every mismatch and deletion, the score will be reduced by 3, i.e. 10-3=7. But for insertion, the score is reduced by one i.e. 10-1=9. So to sort out this problem, the algorithm uses the score as -3 for only insertion. Then, score for insertion (3^rd^ instance) will be 10-3=7. Thus, imbalance is maintained and one can know that the total score is reduced by 3 for every mutation.

The alignment matrix is constructed by taking the query sequence along the top and the motif pattern down the page. The backtracking to find the motifs is done by using a simple logic. Let us consider a motif of length n. Thus, the scores for different mutations will be as follows:

For 0 mutation: 2(n-0) – 1×0 = 2n.
For 1 mutation: 2(n-1) – 1×1 = 2n – 3.
For 2 mutation: 2(n-2) – 1×2 = 2n – 6.
For 3 mutation: 2(n-3) – 1×3 = 2n – 9.
And so on

Finally if m is the number of mutations, then the score will be 2n-3×m. So directly, one can know that the end point of different motif instances, as mentioned above where every scores are 2n-3×m, then it will be end point of motif instance with m mutations. It is possible to trace the motif by the backtracing technique. By using this, one can find the end point of the various instances of the motif with a given number of mutations and also trace the whole motif with the mutation. Here, the tracking of motifs is done by giving preference to those with fewer numbers of mutations (starting with no mutations).The algorithm begins by tracking those motifs with zero mutations (i.e. in the last row the position with score 2n) and by blocking those regions in the query sequence to restrict the next iteration from searching the same regions again to avoid overlapping and reduce time consumption. Then, in the next iteration, it searches for motifs with mutation (numbers are increasing with each iteration) in the unblocked regions of the query sequence and trace back. If it overlaps with the blocked region, then they are ignored else they are accepted. Finally, all motifs are displayed in color starting and ending with “*”, and all mutations are displayed with different colors and indications.

### Study 2: Alignment matrix with different numbers of mutations

**Fig. 2:**
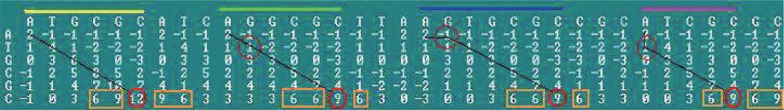
Alignment matrix with different numbers of mutations.

Alignment matrix with different numbers of mutations is explained in ***Fig. 2***.

Sequence:
ATGCTCATTTAATCACAGGCCTGCTCAAATTTGTTCAAAGGTGCTCATTTTTTTAATCAAA

Motif: TGCTCA
Number of allowed mutations: 2.
→1^st^ iteration for exact matches: yellow, score will be 12 i.e. 2n

**Figure:**
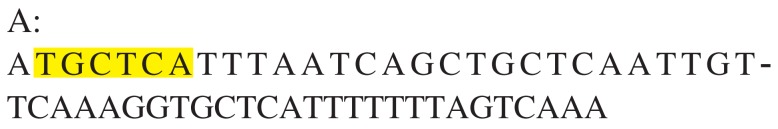


All these positions are blocked, which means that the next instance cannot overlap this instance. Next iteration will search for instance in unblocked (or uncolored) region.

**Figure:**
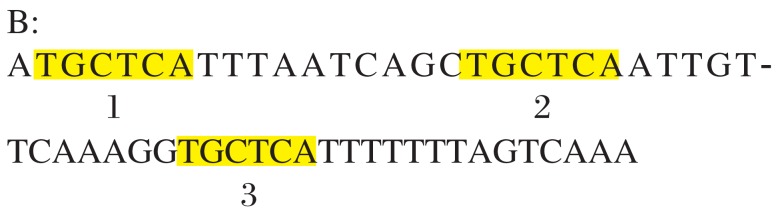


Thus, all instances with zero mutations are discovered and blocked for the next iteration so that not to search in those areas.

→2^nd^ iteration for instances with 1 mutation: green, score will be 9 or 2n-3

**Figure:**
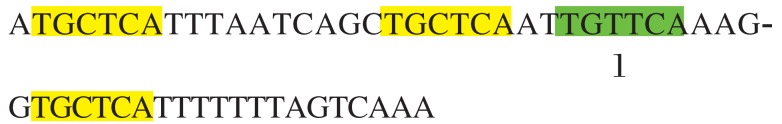


→3^rd^ iteration for instances with two mutations: pink, score will be 6 or 2n-6

**Figure:**
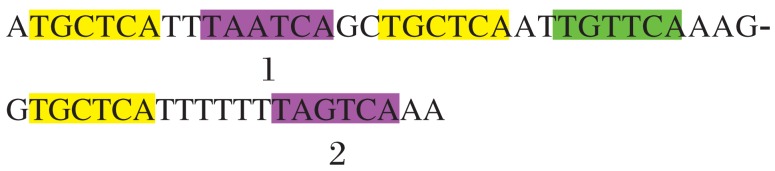


Here, we calculated alignment matrix between the above given sequence (up to 33 nt only) and motif. In the above matrix the last row scores give us the idea of motif occurrences. Most values in the last row will be of the form 2n – 3m. After calculation of the alignment matrix, the further steps will be as follows: 1) Here motif length is 6, so the maximum score will be 12, i.e. for exact match. So for zero mutation motif occurrences, we will search for 12 in the last row, then from the position where 12 is found we backtrack to the first row as shown above. Finally, we block that region (shown with yellow color) and search for next occurrence of 12 and repeat the same. If second instance happens to overlap the already blocked region, we ignore the current occurrence and go for next. 2) For motif with one mutation, the score will be 9, and we search for 9 in the last row and we back track from that position if it overlaps the already blocked regions. We ignore that and go for the next. Positions with orange color enclosed are ignored due to overlapping. 3) Similarly, for motif with two mutations, we search for 6. Finally, we get all possible motif occurrences with different mutations.

## DISCUSSION

AMF is designed as a tool controlled by the PHP and IIS server. These typescripts mediate interaction with the user, the tool and the web server. The tool component of AMF is implemented with the IIS server on a Solaris 10 operating system (QUAD CORE Xeon processor). The PHP script was used for designing and validating, respectively, the input page. The proposed tool is very user friendly and has been tested extensively on various computing platforms using the Mozilla Firefox browser. The tool is validated and its response time is very fast. Of course, the response time may vary depending upon the network speed and the number of users accessing the tool at that particular time.

In conclusion, AMF is a web-based framework to discover DNA motifs through a minimal setup of the tools on the server. Our evaluations show that it can detect all periodic patterns, which are not easily discovered using a sequential approach. This comprehensive knowledge resource will be of great use to researchers working in the area of biotechnology, computational chemistry and bioinformatics. This biotool will help to improve our understanding of the architecture of different mutation based motifs in DNA sequence. This tool can be freely accessed at http://www.sastra.edu/scbt/amf/. The users of this tool are requested to cite this article in their scientific reports. General comments and suggestions for additional options are welcome and should be sent to the corresponding author, R. Seenivasagam (e-mail: seenivasagam.pharma@gmail.com).
